# Adapter-based allogeneic CAR T cells to overcome antigen escape in solid tumors

**DOI:** 10.3389/fimmu.2026.1822583

**Published:** 2026-04-22

**Authors:** Phuc Q. Pham, Quaovi H. Sodji

**Affiliations:** 1 Department of Human Oncology, University of Wisconsin-Madison, Madison, WI, United States; 2 Cellular and Molecular Pathology Program, University of Wisconsin-Madison, Madison, WI, United States; 3 Carbone Cancer Center, University of Wisconsin-Madison, Madison, WI, United States

**Keywords:** allogeneic cell therapy, antigen escape, CAR T cell therapy, CRISPR/Cas9, T cell receptor, tumor-associated antigen

## Abstract

Monospecific Chimeric Antigen Receptor (CAR) T cell therapy against hematological malignancies targeting specific tumor-associated antigen (TAA) has gained clinical success in recent years. Despite their clinical outcomes, challenges including antigen escape, time and labor-intensive manufacturing process, and diminished efficacy especially against solid tumors persist. While allogeneic monospecific “off-the-shelf” CAR T cell therapy from healthy donors with knockout of alloreactive genes using gene editing tools such as CRISPR/Cas9 or TALEN has been evaluated to overcome manufacturing challenges, these allogeneic CAR T cells still face antigen escape. As such, adapter-based CAR T cells that can be redirected by small-molecule adapters to target multiple TAAs have emerged as an alternative therapeutic platform to overcome antigen escape. However, autologous adapter-based CAR T cell manufacturing remains time and labor intensive and scales poorly. Furthermore, chemotherapy-induced T cell dysfunction may compromise both manufacturing and efficacy of autologous CAR T cells. In this comprehensive review, we highlight advantages and limitations of the adapter-based CAR T platform and discuss how allogeneic manufacturing can be applied to adapter-based CAR T as a potential “off-the-shelf” therapeutic for treating multiple cancer types and overcome antigen escape.

## Graphical Abstract

**Figure:**
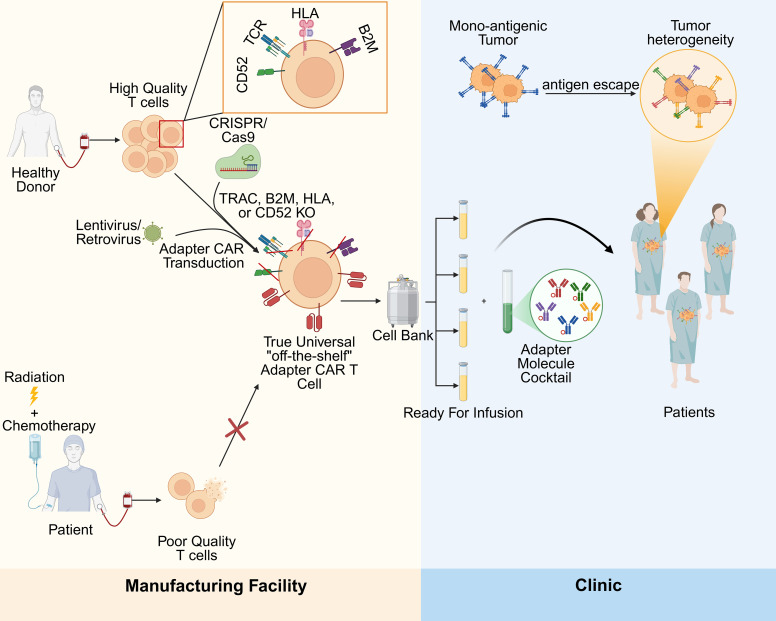

## 1 Introduction

**Figure 1:**
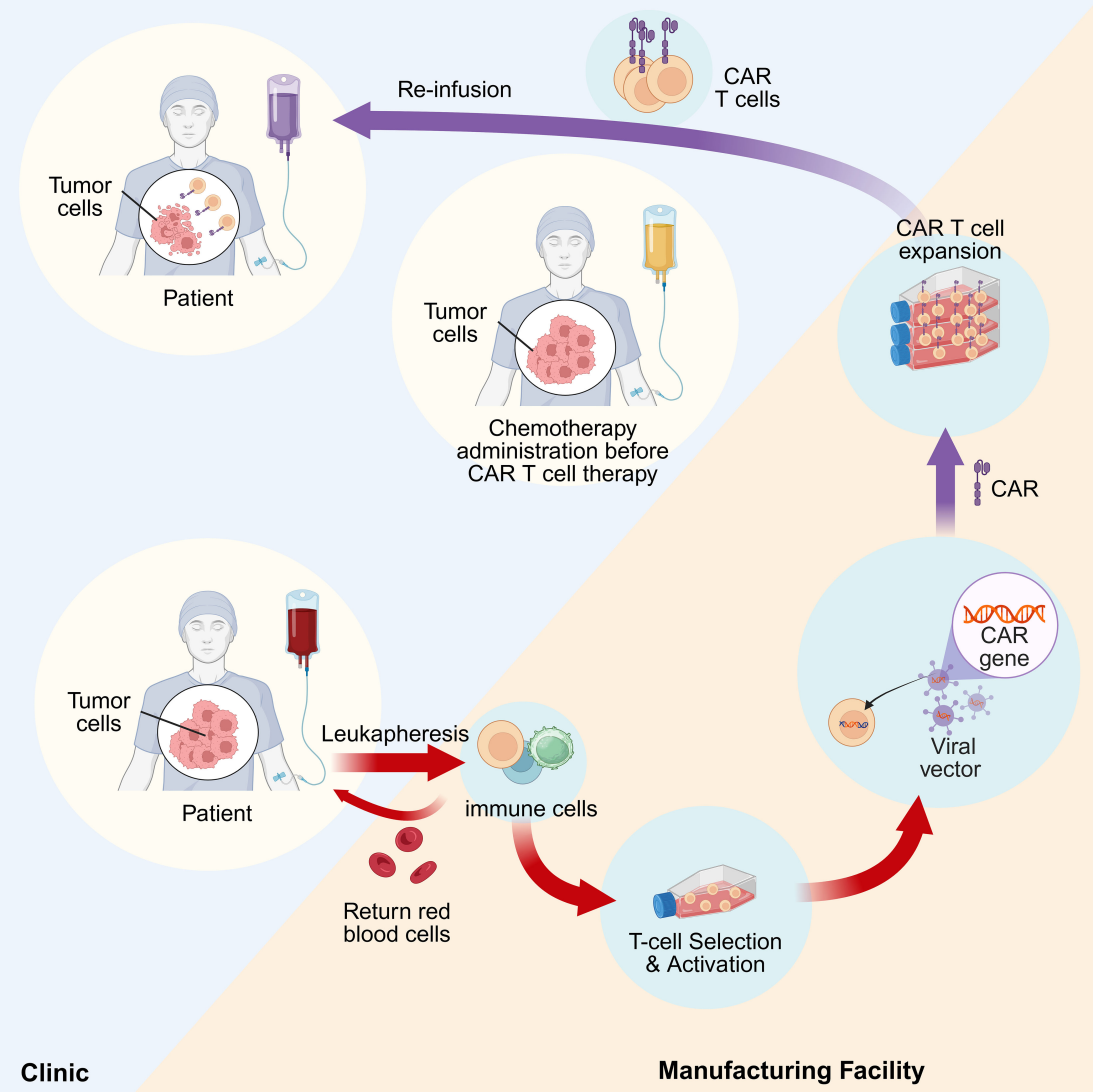
Steps in the manufacturing of autologous CAR T cells. Leukapheresis is employed to collect peripheral blood and isolate T cells from patients. Following T cell activation, the chimeric antigen receptor (CAR) transgene is introduced *ex vivo* via genetic modification to generate CAR T cells. The CAR T cells are subsequently expanded and then re-infused into the patient for therapeutic application.

Adoptive T cell therapies including tumor-infiltrating lymphocyte (TIL) therapy, engineered T cell receptor (TCR) therapy and chimeric antigen receptor (CAR) T cell therapy are currently being developed as therapeutic options for cancer. Both engineered TCR therapy and CAR T cell therapy involve the transfer of genetic materials to T cells to produce transgenic receptors targeting specific tumor antigen (1–6). Various transgene delivery methods such as viral vectors, electroporation (EP), and lipid nanoparticles (LNPs) have been extensively evaluated for CAR T cell manufacturing, with a focus on enhancing anticancer activity, CAR T cell persistence, and minimizing toxicities including cytokine release syndrome (CRS) and secondary malignancies. Monospecific CAR T cell therapies using gene editing tools such as CRIPSR/Cas9 or TALEN to knock out the TCR, CD52, and PD-1, or overexpress CD47, have shown reduced toxicity and enhanced efficacy in clinical trials (7–11). All seven FDA-approved and EMA-approved CAR T cell products are monospecific, virally transduced from autologous T cells and have demonstrated excellent clinical outcomes, including pediatric and adult leukemias (12–15) (**Figure 1**; **Table 1**).

**Table 1:** FDA-approved CAR T cell products.

CAR-T product	Year of approval	CAR generation/Co-stimulatory domain	Targeted antigen	Clinical indications
Tisagenlecleucel (Kymriah^®^)	2017	Second generation 4-1BB	CD19	• Pediatric and young adults up to 25 y with relapsed or refractory B-ALL. • Adult patients with relapsed or refractory large B-cell lymphoma and follicular lymphoma after 2 or more treatment lines
Axicabtagene ciloleucel (Yescarta^®^)	2017	Second generation CD28	CD19	• Adult patients with relapsed or refractory large B-cell lymphoma and follicular lymphoma after 2 or more treatment lines
Brexucabtagene autoleucel (Tecartus^®^)	2020	Second generation 4-1BB	CD19	• Adult patients with relapsed or refractory mantle cell lymphoma. • Adult patients up to 26 y with relapsed or refractory B-ALL
Lisocabtagene maraleucel (Breyanzi^®^)	2021	Second generation 4-1BB	CD19	• Adult patients with relapsed or refractory large B-cell lymphoma, chronic lymphocytic leukemia, and small lymphocytic lymphoma after 2 or more treatment lines
Idecabtagene vicleucel (Abecma^®^)	2021	Second generation 4-1BB	BCMA	• Adult patients with relapsed or refractory multiple myeloma after 2 or more treatment lines
Ciltacabtagene autoleucel (Carvykti^®^)	2022	Second generation 4-1BB	BCMA	• Adult patients with relapsed or refractory multiple myeloma after 1 or more treatment lines
Obecabtagene autoleucel (Aucatzyl^®^)	2024	Second generation 4-1BB	CD19	• Adult patients with relapsed or refractory B-ALL

B-ALL, B-cell Acute Lymphoblastic Leukemia; BCMA, B-cell maturation antigen.

Despite advances in autologous CAR T cell therapy, manufacturing failure occurs in up to 13% with CD19-targeting CAR (16) and 8% with B-cell maturation antigen (BCMA)-targeting CAR (17). Moreover, prior radiotherapy or cytotoxic chemotherapy often causes lymphopenia, limiting the availability of T cells for autologous CAR T cell manufacturing (18, 19). To address this limitation, allogeneic CAR T cells derived from healthy donors offer an “off-the-shelf” alternative, expanding the clinical potential of CAR T cell therapy (20, 21). These allogeneic CAR T cells (allo-CAR), also referred to as “universal” CAR T cells, have demonstrated promising results in clinical trials (**Table 2**). However, while they mitigate the manufacturing challenges associated with autologous CAR T cell therapies, they remain susceptible to antigen escape due to their monospecific nature (25). Adapter-based CAR T cells are also known as modular or switchable CAR T cells, where CAR T cells target a small molecule conjugated to an antibody directed against a specific tumor antigen. This adapter-based CAR T cell therapy has been evaluated in clinical trials using CAR T cells derived from autologous T cells (**Table 3**). Although these adapter-based CAR T cells can mitigate antigen escape by utilizing a cocktail of small molecule-labelled antibodies directed against multiple antigens, they still face challenge associated with autologous CAR T cell manufacturing.

**Table 2:** Selected allogeneic monospecific CAR T clinical trials.

CAR construct delivery method	Gene editing method	Target	CAR construct	Indication	Responses/ref/NCT#
Lentivirus	CRISPR (TRAC and CD52 KO)	CD19	Anti-CD19 CAR/4-1BB/CD3-ζ with TRAC and CD52 KO	R/R B-ALL Children	33% MRD- CR (8) #NCT04557436
Lentivirus	TALEN (TRAC and CD52 KO)	CD19	Anti-CD19 CAR with TRAC and CD52 KO	(R/R) (B-ALL) Young adults and adults	CR 48% CR MRD- 36% (NCT02746952) (9)
non-viral transposon-based integration (piggyBac^®^ DNA Delivery System)		MUC1-C	anti-MUC1-C CAR with T cell receptor beta chain 1 (TRBC1) and β2-microglobulin KO and iCasp9 suicide gene	advanced or metastatic epithelial-derived cancers (esophageal adenocarcinoma, colorectal adenocarcinoma, and breast cancer)	Ongoing (NCT05239143) (22)
Lentivirus	CRISPR (HLA I/II KO and CD47 overexpression)	CD19	anti-CD19 CAR with depletion of HLA class I/II and overexpression of CD47 (SC291)	(R/R) B-cell malignancies Adults	CR 0% (NCT05878184) (7)
Lentivirus	CRISPR (TRAC, TRBC, B2M KO)	CD19	anti-CD19 CAR/41BB/CD3- ζ with TRAC, TRBC, B2M-KO	(R/R) CD19+ B-cell leukemia or lymphoma Child, Young adults, and Adults	N/A (NCT03166878) (NCT03229876)
Lentivirus	Codon-optimized cytidine base editor (coBE) mRNA CRISPR (CD52, CD7, and TRAC KO)	CD7	Anti-CD7 CAR/4-1BB/CD3z with CD52, CD7, and TRAC KO	(R/R) ALL Children and Adults	CR 64% (NCT05397184) (23)
Lentivirus	CRISPR (CD7, HLA-II, and TRAC KO)	CD7	Anti-CD7 CAR/4-1BB/CD3z with CD7, HLA-II, and TRAC KO	(R/R) AML or ALL Children and Adults	CR MRD- 60% (NCT04599556) (NCT04538599) (24)

AML, Acute myeloid leukemia; B-ALL, B-cell Acute Lymphoblastic Leukemia; CR, complete response; CRISPR, Clustered Regularly Interspaced Short Palindromic Repeats; HLA, Human Leukocyte Antigens; KO, Knockout; MRD, Measurable Residual Disease; R/R, relapsed and refractory; TALEN, Transcription activator-like effector nuclease; TRAC, T-cell receptor alpha constant; TRBC, T-cell constant β chain.

**Table 3:** Selected autologous adapter-based CAR T clinical trials.

CAR construct delivery method	Target	Adapter	CAR construct	Indication	Responses/ref/NCT#
y-Retrovirus	CD20	Rituximab	anti-rituximab CD16V-Fc CAR/CD28/CD3- ζ	(R/R) CD20+ B cell lymphoma (NHL) Adults	CR 40-50% (NCT03189836) (26) (NCT02776813) (27)
Lentivirus	CD19	SWI019 adapter: anti-CD19 Fab linked to GCN4 peptide neo-epitope (PNE)	anti-GCN4 CAR	(R/R) B-cell malignancies Adults	CR 67% (NCT04450069) (28)
Lentivirus	CD123	TM123 adapter: CD123 Target Module	anti-TM123 CAR/CD28/CD3- ζ	(R/R) AML Adults	CR 0% (NCT04230265) (29, 30)
N/A	CLDN18.2	anti-CLDN18.2 antibody with the P329G mutation	anti-P329G CAR	CLDN18.2+ advanced gastric or pancreatic cancer Adults	CR 0% (NCT05199519) (31)
Lentivirus	CD123	anti-CD123 adapter with a TAG region (SPRX002)	anti-TAG CAR/41BB/CD3- ζ	(R/R) AML or high-risk myelodysplastic syndrome (MDS)	Ongoing (NCT05457010) (AML)

AML Acute myeloid leukemia; CLDN18.2, Claudin 18 isoform 2; CR, complete response; KO, Knockout; NHL, Non-Hodgkin lymphoma; R/R, Relapsed and Refractory; TRAC, T-cell receptor alpha constant.

To our knowledge, while allogeneic CAR T cell therapy (6) and adapter-based CAR T cell therapy (32) have extensively covered elsewhere, no review offers a unified integrative framework of both strategies. Therefore, this review outlines current progress and limitations in monospecific CAR T cell therapies, with a focus on adapter-based platforms, and explores how allogeneic manufacturing could enable truly “universal” CAR T cells that address both antigen escape and autologous cell manufacturing constraints.

## 2 Structure of T cell receptors and chimeric antigen receptors.

**Figure 2:**
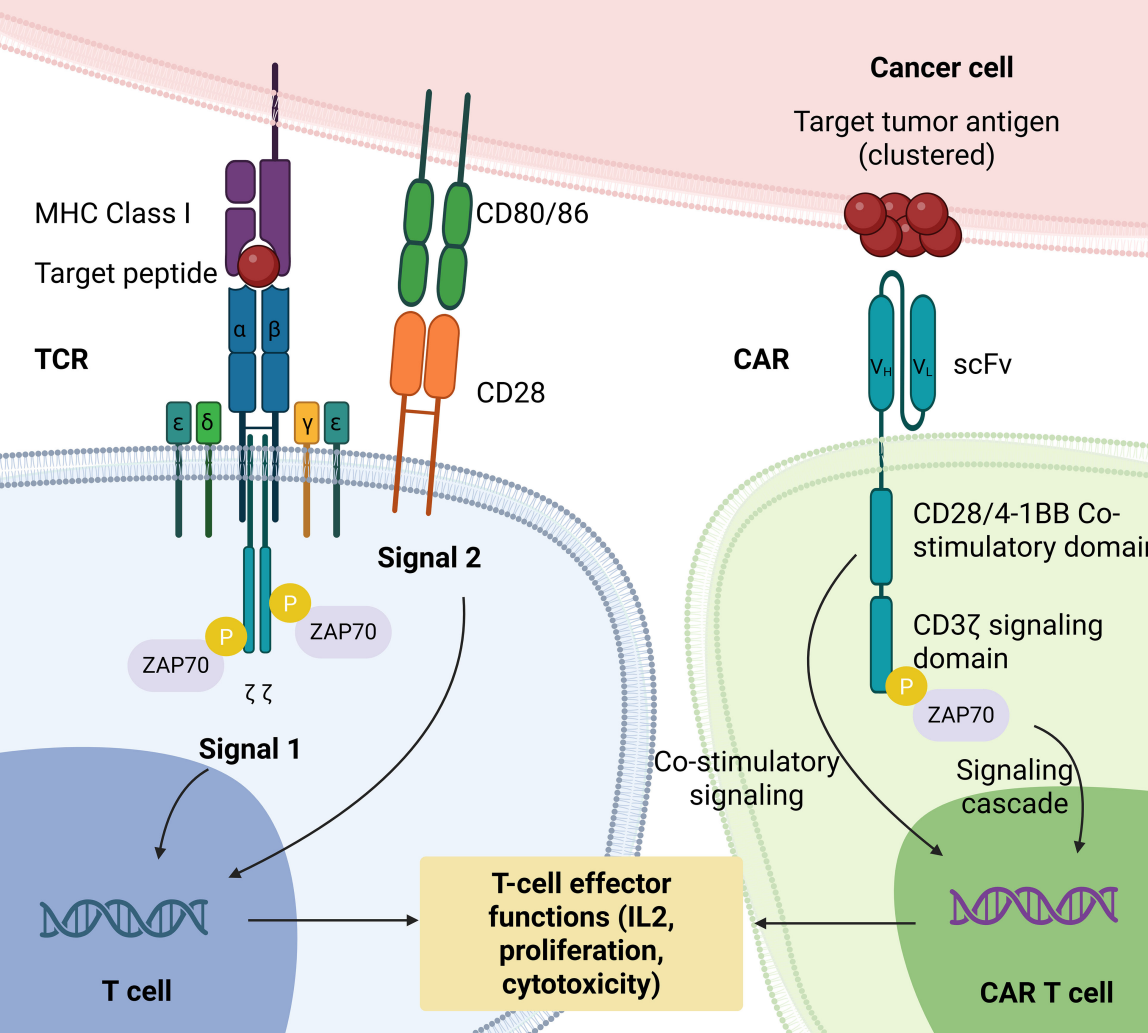
Comparison of TCR and CAR activation. T cells require the presentation of tumor-associated antigens (TAAs) via major histocompatibility complex (MHC) molecules and depend on both antigen recognition and co-stimulatory signals for complete activation. In contrast, CAR T cells (second-generation CAR or higher) are capable of recognizing TAAs independently of MHC presentation and can achieve full activation solely through antigen binding.

T cell receptors (TCRs) are heterodimeric proteins composed of α and β chains that recognize antigens presented by antigen-presenting cells (APCs), including tumor cells, through major histocompatibility complex (MHC) class I or II molecules. These chains are associated with CD3 subunits δ, ε, and γ, as well as signaling ζ chains, which collectively mediate intracellular signaling (33–36). Upon antigen engagement, TCRs initiate a primary recognition signal (signal 1), while full T cell activation and effector function require a secondary co-stimulatory signal (signal 2), typically mediated by the interaction of CD28 on the T cells with CD80 and CD86 ligands on APCs (**Figure 2**).

**Figure 3:**
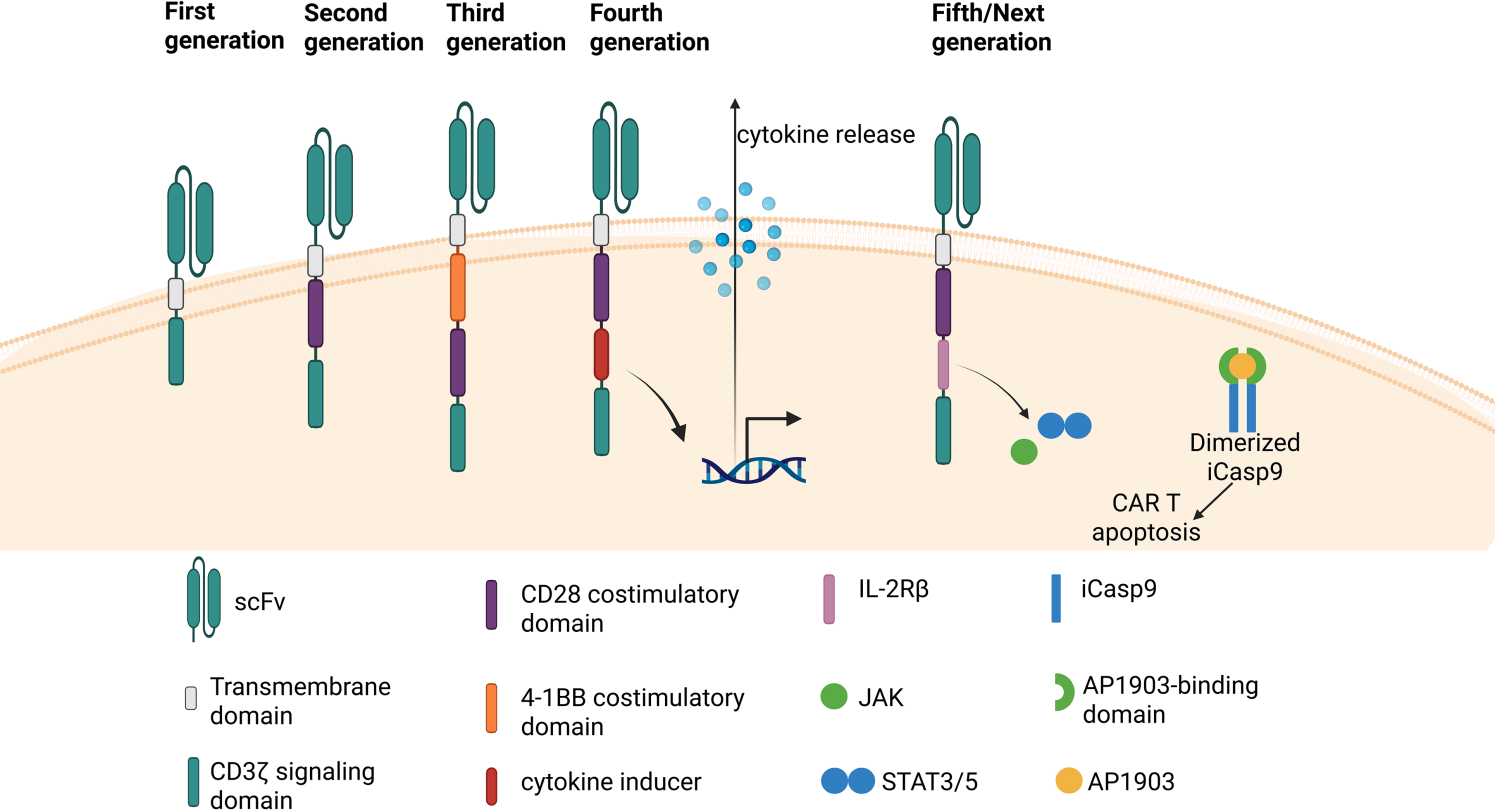
Schematic of all generations of CAR. Generations of monospecific CAR T cell therapies have been developed. First generation CARs have the ScFv, Hinge, Transmembrane, and CD3ζ domains. Second generation CARs have the additional co-stimulatory domain. Third generation CARs have two co-stimulatory domains. Fourth generation CARs are like second generation CARs with the additional cytokine inducer domain. Fifth/Next generation CARs are also based on second generation CARs but include the additional domain of cytokine receptor IL-2Rß and a transcription factor binding domain STAT-3/5, with the optional AP1903-dependent ON-and-OFF-switch in some versions.

Chimeric antigen receptors (CARs) represent a distinct class of engineered receptors that do not require antigen presentation via MHC complex (37). Structurally, a CAR comprises three key components: (1) intracellular signaling domains, including a T cell activation domain and co-stimulatory domain (second generation CAR or higher); (2) a hinge-transmembrane region; and (3) an antibody-derived extracellular binding domain that recognizes specific antigens (6, 37) (**Figure 2**). Over the past decades, multiple generations of CARs have been designed. First-generation CARs were developed using single-chain variable fragments (scFvs) as the extracellular domain but lacked co-stimulatory domains (38–40). To enhance T cell persistence and potency, second-generation CARs incorporating a single co-stimulatory domain CD28 (41) or 4-1BB (42–46) were developed (**Figure 3**). Third generation CAR expanded on the aforementioned design by incorporating an additional co-stimulatory domain, yielding CAR with dual co-stimulatory domains. Fourth-generation CARs which is based on the second-generation CAR design but includes an additional intracellular domain, a cytokine inducer such as interleukin 12 (IL12) which is induced upon CAR activation have also been developed and tested in clinical settings (47–49) (**Figure 3**). Fifth-generation, also known as next-generation or armored, CARs represent one of the most recent iterations of CAR T cell therapy, with the goal to enhance therapeutic efficacy, persistence and address safety issues noted with previous generations. It includes a truncated intracellular domain of cytokine receptor (IL-2Rß) and a transcription factor binding domain such as STAT-3/5. Additionally, they can incorporate a drug-dependent ON-and OFF-switch to further regulate CAR T cell activation or depletion (50). These CAR T cells are currently undergoing clinical investigation and are demonstrating encouraging results (51–53) (**Figure 3**).

Unlike TCRs, CARs recognize antigens independently of MHC presentation, thereby circumventing tumor-induced MHC downregulation, a mechanism of resistance to TCR-dependent T cell activation (**Table 4**). However, CAR T cells targeting is limited to cell surface antigens expressed on the cell surface (6, 54). CARs also need 100-fold more antigens to activate compared to their native TCRs (56). Nonetheless, CARs offer a broader antigen recognition profile by targeting also non-protein antigens, including carbohydrates or glycolipids (55).

**Table 4:** Comparison between T cell receptor (TCR) and chimeric antigen receptor (CAR).

Factors	TCR	CAR
Receptor structure	T-cell receptor heterodimer (35)	Single-chain variable fragment (scFv) from antibody (1)
Target antigen	Peptides presented by MHC complex (33, 34)	Cell surface antigens (proteins, carbohydrates, glycolipid) (37, 54, 55)
MHC dependence	Yes	No
Target types	Intracellular/Surface peptides (5, 54)	Surface antigens (4)
Co-stimulatory signal dependence	Yes	No (41, 42)
Minimum number of antigens required on target cells	1-10 (2)	>100 (56)
Serial killing	Yes (1)	Yes (5)
Challenges	MHC downregulation (6)	Surface antigen downregulation (15)
Toxicity	Autoimmunity (2, 6)	CRS, On-target-off tumor, ICANS (7, 9)

CRS, Cytokine Release Syndrome; ICANS, Immune Effector Cell-Associated Neurotoxicity Syndrome; MHC, Major Histocompatibility Complex.

## 3 Tumor antigen escape as a mechanism of resistance to CAR T cell therapy

CAR T cell therapy in solid tumors faces key barriers including limited infiltration and trafficking in an immunosuppressive TME, and on-target/off-tumor toxicity. While these have been reviewed elsewhere (57, 58), herein we focus on antigen escape in solid tumors.

### 3.1 Tumor antigen escape to monospecific CAR T cell therapy

Early clinical trials of CD19-directed CAR T cells in B-cell acute lymphoblastic leukemia (B-ALL) reported response rates up to 90% in children and young adults (59–61) and showed efficacy in adults with relapsed/refractory B-cell leukemias (62–64). Despite the successes of these FDA-approved CD19 CAR T cells, tumor resistance and disease relapse remain key challenges (14, 15, 65). Loss or mutation of the targeted antigens enables tumors to evade CAR T cell killing. Up to 25% of pediatric complete responders relapse with CD19-negative disease after CD19 CAR T cell therapy (15, 59, 61, 66). Although CD19-negative disease relapse has been observed in adults, these relapses are less frequent than in pediatrics (62, 67). Antigen escape has also been reported after BCMA-targeted CAR T cells in multiple myeloma (68) and IL13Rα2- or EGFRvIII-targeted CAR T cells in glioblastoma (69, 70). Collectively, these data indicate that antigen escape is a key driver of relapse after CAR T cell therapy and highlight the need for strategies to prevent or overcome such resistance mechanism.

### 3.2 Bispecific CAR T cells to overcome limitations of monospecific CAR T cells

**Figure 4:**
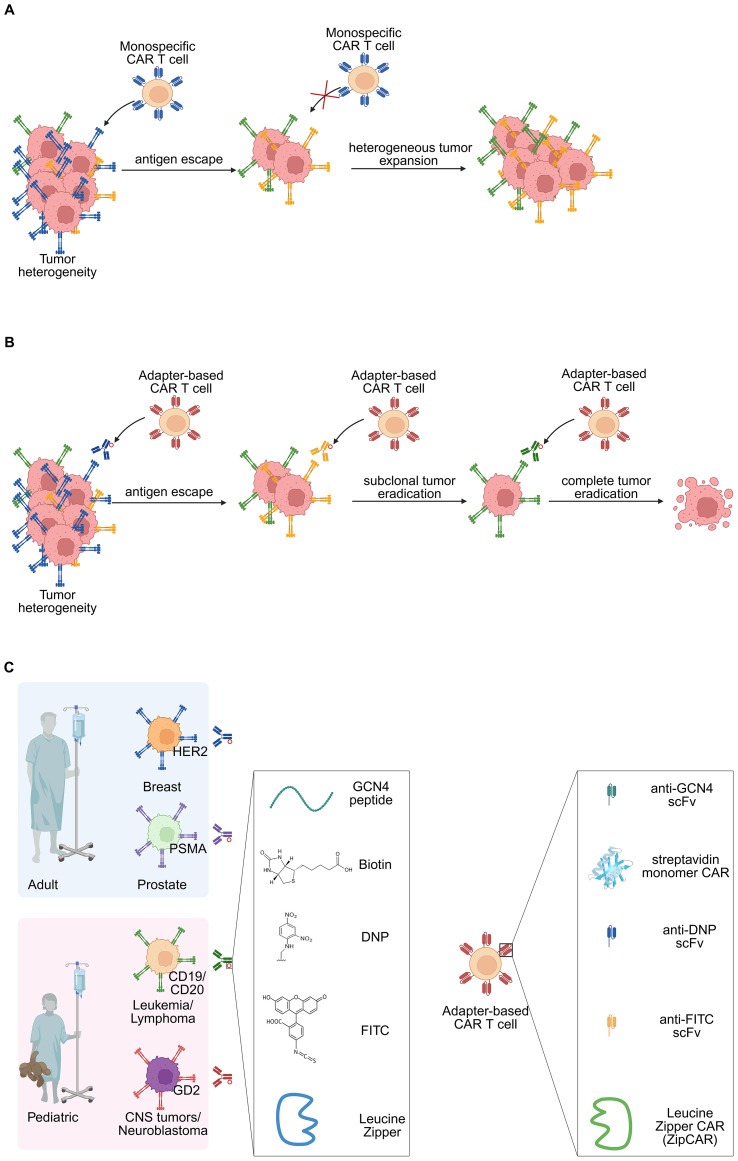
Adapter-based CAR T cell therapy has potential to circumvent tumor heterogeneity and antigen escape issues. **(A)** Monospecific CAR T cell therapy faces the challenge of antigen escape that tumor cells downregulate the target TAA and become antigenically heterogeneous. **(B)** Anti-small-molecule CAR T cells can target multiple TAAs with the redirection of small-molecule-labeled antibodies function as adapters. **(C)** These small molecules, including biotin, DNP, FITC, GCN4 peptide, and leucine zipper, can be tagged onto different antibodies to target the corresponding surface antigens. Well-studied TAAs of some common adult cancers include HER2 (breast) and PSMA (prostate), and pediatric cancers include CD19/CD20 (leukemia/lymphoma) and GD2 (CNS tumors/neuroblastoma).

Despite early successes, single-target CAR T cell therapy has some limitations. First, monospecific CAR T cells are vulnerable to antigen escape via target downregulation or mutation (**Figure 4A**). Second, inter- and intratumoral antigen heterogeneity which can result in differential antigen expression, yielding inconsistent response to CAR T cell therapy (71). To address this, dual/bispecific CAR T cells directed against two tumor antigens have been developed. Bispecific CAR T cells targeting CD19/CD22 (72–74) or CD19/CD20 (75–77) have shown promising activity in relapsed/refractory B cell malignancies, yet resistance still arises as the selection pressure by these CAR T cells drives antigen modulation. Moreover, these bispecific targeting approaches are largely confined to leukemias expressing CD19, CD20 or CD22 and have not broadly translated to solid tumors, where clinical effectiveness remains to be established.

Although CAR T cell therapies for solid tumors have been extensively studied, their clinical applications remain very limited for various reasons (78–80). Examples of antigens clinically targeted by monospecific CAR T cells include the disialoganglioside GD2 in neuroblastoma (81) and mesothelin (MSLN) which is overexpressed in plethora of solid tumors (82). Fibroblast activation protein (FAP), overexpressed on cancer-associated fibroblasts (CAFs) and tumors is another putative broad target in solid tumors (83, 84). Targeting broadly shared antigens (e.g. MSLN, FAP) by solid tumors is promising when it comes to the development of an effective monospecific CAR T cell therapy against solid tumors. However, these CAR T cells remain susceptible to antigen escape. To address both tumor cells and immunosuppressive tumor microenvironment (TME) targeting, MSLN CAR T cells secreting a T cell-engaging agent directed towards CAF have been tested in pancreatic ductal adenocarcinoma (PDAC) with improved activity over MSLN CAR T cells alone, suggesting the superiority of a dual targeting approach in solid tumors (85). As in leukemia, antigen escape and heterogeneric antigen expression can still drive resistance in solid tumors. For example, a PSCA/MUC1 bispecific CAR T cells in prostate cancer achieved greater cytotoxicity than either monospecific CAR T cells, yet low expression of both antigens enabled resistance to the bispecific CAR T cells (86). Thus, broadening antigen coverage is therefore a key focus of ongoing therapeutic development.

### 3.3 Multi-specific CAR T cells to enhance the efficacy of CAR T cell therapy

A trispecific CAR T cell therapy targeting EGFRvIII, EphA2, and HER2 outperformed mono and bispecific CAR T cells in patient-derived xenografts models (87). It is undeniable that targeting multiple antigens mitigates the potential risk of antigen escape ensuing from a monospecific CAR T cell therapy. However, manufacturing a separate monospecific CAR T cell for each tumor-associated antigen is impractical and multi-specific CAR designs support simultaneously only a limited set of antigens. Potential challenges include the development, testing and regulatory approval of each CAR construct which is resource and time consuming. Furthermore, combination regimens contribute to toxicity requiring additional testing. Optimizing antigen combinations for multi-specific CAR T cell therapies to target diverse cancer types is also an obstacle yet to be fully circumvented. Moreover, expanding the number of targeted TAAs reaches safety limits, as many antigens are shared with normal tissues, increasing the risk of on-target, off-tumor effects. These constraints underscore the urgent need for universal, multi-TAA CAR T cell strategies that curb antigen escape while improving scalability, regulatory regulation and validation and cost effectiveness.

## 4 Adapter-based autologous CAR T cell therapy: using small molecule-conjugated anti-TAA antibodies as adapter molecules

To circumvent the aforementioned obstacles facing mono- and bispecific CAR T cells, adapter-based CAR T cells have been developed and whose activity is redirected by the concurrent administration of adapters targeting multiple TAAs (88–91). Unlike conventional CAR T cell therapy, the scFv is designed to recognize a chemical moiety such as a small molecule bound to an adapter, instead the specific TAA (**Figure 4**). This section summarizes the various adapter-based CAR T cell therapies and addresses their cytotoxicity against multiple cancers and TAAs without requiring new CAR T cells engineering.

### 4.1 Biotin-based adapters

Biotin-binding immune receptor (BBIR) CAR T cells retain second-generation signaling and hinge domains but replace the conventional scFv with an avidin extracellular domain (AED) that binds biotinylated antibodies/adapters (88). Swapping antibodies/adapters redirects BBIR CAR T cells to multiple TAAs. In preclinical models, BBIR CAR T cells were successfully redirected with biotinylated anti-EPCAM, anti-mesothelin, and anti-folate binding protein/FRα antibodies to target tumors expressing the corresponding TAA expression, whereas non-biotinylated antibodies were inactive, confirming biotinylated adapter-dependent specificity (88). Physiological or elevated biotin neither displaced the labelled antibodies/adapters nor activated the BBIR CAR, indicating low risk of endogenous biotin activation. Because biotin is a human vitamin (92), BBIR CAR T cells have clinical potential with minimal risk of TAA-independent activation by endogenous molecules. The monomeric streptavidin mSA2 CAR binds biotin with affinity comparable to that of dimeric BBIR CARs (93, 94) and when stimulated by plate-immobilized biotin and co-administered with biotinylated cetuximab (anti-EGFR), rituximab (anti-CD20), or FMC63 (anti-CD19) mediates cytotoxicity against corresponding tumors. Site specificity of biotinylation of adapter is likely critical, whereas enzymatic/site-directed methods control biotin position, N-hydroxysuccinimide (NHS) chemistry is fast and inexpensive but yields random conjugation sites and stoichiometry, potentially perturbing function and avidity (95,) (94). Because adapters increase the CAR immune-synapse gap, precise site of biotin conjugation is functionally important (4). Overall, the AED serves as a scaffold allowing multiple biotinylated antibodies to redirect BBIR CAR T cells to tumors, and without biotinylated adapters, the BBIR CAR T cells remain inactive, limiting off-target, antigen-independent cytotoxicity. While this platform leverages the well-established biotin/streptavidin interaction (96, 97), potential pitfalls remain including interference by endogenous biotinylated proteins and immunogenicity of streptavidin CAR extracellular domains and biotinylated adapters (98).

### 4.2 GCN4-based adapters

Some adapter-based CAR uses an anti-GCN4 scFv to generate a second-generation CAR T cell therapy (99). This anti-GCN4 scFv recognizes a 14-amino-acid peptide from yeast GCN4, a transcription factor absent in humans, and predicted to be weakly immunogenic, allowing GCN4-fused antibody adapters to redirect a single CAR to multiple TAAs with minimal cross-reactivity (99). This approach has been successfully utilized with the CD19 antibody FMC63 and CD20 antibody ofatumumab to redirect CAR T cells towards B cell tumors (89). The GCN4 peptide placement on the adapters/antibodies modulated the subsequent CAR T cell activity: with the anti-CD19 FMC63 adapter, N-terminal bivalent tagging with GCN4 increased cytokine release compared to the C-terminus tagging, highlighting the importance of the immunological synapse geometry in CAR function (89, 100). Conversely, with a shortened IgG4 hinge, N-terminal tagging reduced the potency of the anti-CD20 antibody ofatumumab-based adapter (89). This suggests that optimizing this CAR targeting approach requires multiple considerations including the CAR construct, adapter structure, peptide fusion sites, and the distance between the cell membrane and the targeted antigen, all of which impact the immune synapse formation and consequently CAR T cell potency. In Nalm-6 xenografts, CAR T cells with a N-terminus GCN4-tagged FMC63 adapter matched conventional CD19 CAR T cell efficacy (89). CAR T cell trafficking, cytotoxicity and expansion were observed only with the co-administration of GCN4-fused anti-CD19 (FMC63) adapter. Adapter dosing can be titrated by monitoring serum inflammatory cytokines (IL-2 and TNFs), enabling effective tumor killing while minimizing cytokine release, thus potential toxicity (99). These findings highlight the low risks of TAA-independent immunocytotoxicity and CRS when using adapter-based CAR T cells with GCN4-fused anti-CD19 adapters. GCN4-adapter CAR T cells have likewise also been evaluated TNBC mouse models using GCN4-labeled trastuzumab, a FDA-approved, low-immunogenic HER2 antibody (99). Key limitations of the GCN4-based adapter to redirect CAR T cells include the potential immunogenicity of the activators (FMC63) and the GCN4 peptide. Moreover, expanding GNC4-based adapters to target additional TAAs particularly in solid tumors is needed to fully harness the potential benefit of this unique peptide-based approach.

### 4.3 FITC-based adapters

These FITC-based adapters are designed to be utilized with a third-generation CAR T cells endowed with an extracellular domain derived from anti-FITC scFv. This scFv bind to FITC-tagged adapter antibodies such as rituximab (anti-CD20), cetuximab (anti-EGFR), trastuzumab (anti-HER2) and anti-B7H3 thereby redirecting CAR T cells to solid tumors expressing corresponding TAAs (90, 99, 101, 102). Fluorescein isothiocyanate (FITC) is an inexpensive fluorochrome readily coupled to solvent-exposed primary amines via NHS chemistry, typically yielding two FITC molecules per antibody (103–105). Activation of these FITC-targeting CAR T cells, evidenced by expansion, cytokines secretion, cytotoxicity, leading to *in vivo* antitumor response occurred only when co-administered with FITC-tagged antibodies (90). To improve efficacy, specific sites of FITC conjugation were installed using genetically encoded noncanonical amino acids on the CD19 antibody FMC63 and CD22 antibody M971, resulting in a potent dose-dependent antitumor activity in xenograft models (105). Potency was dependent on the site of FITC conjugation: tags proximal to the antigen binding region on the CD19 antibody (G68; S74) were cytotoxic compared to distal labelling sites (S202; K136) (105). Monovalent FITC conjugates were less potent than their bivalent ones *in vitro*. Consistent with these *in vitro* findings, *in vivo* the monovalent antibodies led to tumor relapse (105). These findings highlight the importance of immune synapse stoichiometry and FITC conjugation sites in modulating the potency of FITC-directed CAR T cells. CAR T cell activity and toxicity (cytokine release syndrome, B cell aplasia) can be modulated by controlling the adapter dosing and timing of administration. Another promising solution involves the use of non-targeting FITC-labeled antibodies to compete with the FITC-labeled adapters for the binding to anti-FITC CAR T cells (90).The potential FITC immunogenicity remains a key challenge that must be further evaluated (106) as this platform is entering clinical evaluation (107). While we emphasize herein CAR T cells, this FITC-adapter-based CAR platform can also be applied in other immune cells such as neutrophils and NK cells (108).

### 4.4 Leucine zipper-based adapters

The split, universal, and programmable (SUPRA) CAR system comprises a leucine-zipper receptor expressed on the T cells (zipCAR) and a tumor-targeting scFv adapter (zipFv) (91). The zipCAR encompasses a leucine zipper bound to the intracellular signaling domain, while each zipFv has a TAA-specific linked to a complementary leucine zipper. These coiled-coil leucine zipper pair interactions enable orthogonal competitive and switchable interactions, yielding logic-gated control of CAR T cell cytotoxicity (91, 109–111). SUPRA CAR T cells are activated, resulting in IFN-γ production and subsequent tumor killing only in the presence of zipFv. With this approach, *in vivo*, CRS can be mitigated by administering an inhibitory zipFv at a higher concentration, to outcompete the activating zipFv binding, thereby blocking the zipCAR activation (91). Real-time monitoring of cytokines can enable immediate adjustment of the dosing schedules of inhibitory and activating zipFv to modulate SUPRA CAR T cell cytotoxicity. Moreover, SUPRA CAR T cell potency and toxicity (CRS) can be controlled by modulating zipFv concentrations and leucine zipper components (EE, SYN3, and SYN5) with different affinity to zipCAR (91). Replacing the conventional scFv with a zipper may also reduce CAR T cell exhaustion induced by receptor aggregation (91, 112). Co-administration of zipCAR T cells with zipFvs targeting different TAAs (Axl, HER2) achieved robust killing of malignant cells expressing either antigen (OR-gate), enabling rapid retargeting to address antigen escape without engineering novel CAR T cells (91). In both HER2-positive hematologic and solid tumor models, SUPRA CAR T cells demonstrated tumor control comparable to conventional HER2-directed CAR T cells (91). SUPRA CARs built with orthogonal SYNZIP leucine-zipper pairs can modulate multiple signaling pathways simultaneously (110). In CD4+ T cells, co-expression of two orthogonal SUPRA CARs implemented an AND gate strategy (91): a FOS zipCAR with a CD3ζ signaling domain, recognizes anti-HER2-SYN9 zipFv, while an RR zipCAR with 4-1BB and CD28 co-stimulatory domains, can recognize anti-Axl-EE zipFv. Robust activation occurs only when both CARs engage their respective antigens simultaneously on tumor cell, a strategy validated *in vivo* to demonstrate combinatorial antigen detection (113). In co-culture with both adapters and dual-antigen target cells, the dual-SUPRA-CAR T cells showed synergistic increase in CD69 expression and cytokines including IL-2, IL-4, and IFN-γ (91). This orthogonal design also enables cell subtype-specific modulation, with FOS zipCARs in CD8+ T cells and RR zipCARs in CD4+ T cells which can be activated independently by their respective adapters (91). Beyond CD8+ and CD4+ T cells, SUPRA CARs have been deployed in other immune cells including Treg, γδ T cells, NK cells, and macrophages, underscoring the platform generalizability for engineering diverse immune phenotypes (114).

### 4.5 DNP-based adapters

Adapter-based CARs extend beyond T cells to other immune cells such as NK cells (115). Rather than targeting biotin, FITC or leucine zippers, another approach consists of designing a CAR targeting the small molecule 2,4-dinitrophenyl (DNP). Antibodies labelled with DNP redirect the DNP-directed CAR immune cells towards diverse targets, including HIV-1 gp160 variants and CD19 and CD22, enabling cytotoxicity against HIV-infected cells, Nalm-6 and Raji cells respectively (115, 116). Clinical translation of this targeting approach may be hindered by the fact that ~1% of naturally occurring human antibodies bind DNP, potentially competing with DNP-labelled anti-TAA antibodies/adapters for CAR engagement (117, 118). To address this limitation, anti-DNP CARs should be engineered for higher DNP-binding affinity to enhance the recognition of DNP-labelled adapters. A major hindrance in the clinical use of DNP resides in the fact that it is a mitochondrial uncoupler that disrupts ATP synthesis and can cause hyperthermia (119), with reported acute toxicity including lactic acidosis, electrolyte imbalance (120), as well as teratogenicity, carcinogenicity, and mutagenicity in rats (121). Hence, future adapter-CAR designs could therefore substitute DNP with a less immunogenic small molecule that does not perturb mitochondrial function.

### 4.6 Bioorthogonal click chemistry to redirect CAR T cells toward tumors

Rather than using small-molecule-labeled antibodies to redirect CAR towards malignant cells, metabolic glycan labeling has also been utilized to achieve a similar goal. Azide-functionalized monosaccharides such as N-azidoacetylgalactosamine (GalNAz) and N-azidoacetylmannosamine (ManNAz) are up taken and converted intracellularly to UDP-N-azidoacetylglucosamine (UDP-GlcNAz) and to N-azidoneuraminic acid (SiaNAz), respectively, which are incorporated into cell surface glycans (122–125). This metabolic labeling strategy has been used to label the target cell membrane for many purposes including the delivery *in vivo* of chemotherapeutics and radioisotopes to malignant cells (125–128). The interaction between azide-labelled glycans malignant cells and bicyclononyne (BCN)-labelled immune/engineered cells enables biorthogonal click chemistry, redirecting immune/engineered cells towards malignant cells (126, 129–132). Metabolic glycan labeling with GalNAz/ManNAz or their sialylated derivatives allows conjugation of FITC or DNP, redirecting anti-hapten CAR T cells to malignant cells (133). Despite *in vitro* and *in vivo* validation, labels persist less than forty eight hours, requiring repeated intratumoral dosing (133). Successful implementation of this approach relies on robust tumor cell labeling. Additionally, this approach has been demonstrated in very few tumor histology, suggesting broader testing is needed to confirm applicability across multiple cancer types.

### 4.7 Safety, scalability and translational considerations for adapter-based CAR platforms

#### 4.7.1 Safety

Adapter CAR platforms are inherently complex due to the combination of cell therapy with a separate adapter, raising regulatory burdens. The principal safety concern remains on-target cytotoxicity. The cytotoxicity of these CAR can be abrogated using non-specific antibodies with small-molecules (DNP, FITC, biotin, GCN4), or inhibitory zipFv in the leucine-zipper CAR T cell platform. These non-specific antibodies compete with active adapters for CAR binding, potentially minimizing or abolishing CAR activation-induced toxicity including CRS.

#### 4.7.2 Immunogenicity of adapters

Adapter selection is pivotal for clinical use. Biotin is a vitamin, thus not immunogenic (92), and leucine zipper elements can be humanized to mitigate immunogenicity (91). DNP, in addition to its significant immunogenic risk, is associated with other safety concerns including metabolic disruption, teratogenicity, carcinogenicity and mutagenesis (119–121). Despite immunogenicity concerns, FITC has demonstrated a favorable safety profile and is the only adapter-based CAR platform clinically evaluated to date (107) (**Table 5**).

**Table 5:** Summary of selected adapter-based CAR platforms.

Platform	Adapter recognition domain	Adapter molecule	Disease	Clinical status	Immunogenicity	Pitfalls	Immune cell applications
Biotinylated adapter CAR	Streptavidin monomer CAR	Biotin	Ovarian cancer (88)	Pre-clinical	No* (92, 98)	Competitive inhibition by endogenous biotin or biotinylated proteins	T cells
GCN4-conjugated adapter CAR	Anti-GCN4 scFv	GCN4 peptide	B-cell leukemia and TNBC (89, 99)	Pre-clinical	Yes (89)	Adapter immunogenicity	T cells
FITC-conjugated adapter CAR	Anti-FITC scFv	FITC	B-cell lymphoma, Colon cancer, pancreatic cancer, TNBC and R/R osteosarcoma (90, 99, 102, 107)	Phase I (Active, not recruiting) Start: 05-20-2022 (NCT05312411)	Yes (106)	Adapter immunogenicity	Neutrophils, T cells, and NK cells
Leucine zipper adapter CAR	Leucine Zipper CAR (ZipCAR)	Leucine Zipper	B-cell leukemia and breast cancer (91)	Pre-clinical	No (91)	Understudied clinical applicability	T cells (CD8+, CD4+, Treg, γδ T cells), NK cells, and macrophages
DNP-conjugated adapter CAR	Anti-DNP scFv	DNP	B-cell leukemia (116)	Pre-clinical	Yes (119–121)	Adapter immunogenicity. Limited studies in targeting solid tumors	NK cells and T cells
Metabolic labeling of cancer cells	Anti-DNP or Anti-FITC scFv	DNP- or FITC-conjugated GalNAz, ManNAz, or sialic acids**	TNBC (133)	Pre-clinical	N/A	Unknown immunogenicity of unnatural sugars. Short half-life of FITC or DNP-conjugated glycan	T cells

*Biotin is not immunogenic, but streptavidin CAR and biotinylated adapters are.

**Metabolically labeled on cancer cells.

DNP, 2,4-Dinitrophenol; FITC, Fluorescein isothiocyanate; GalNAz, N-azidoacetylgalactosamine; ManNAz, N-azidoacetylmannosamine; R/R, relapsed and refractory; scFv, single-chain variable fragment; TNBC, triple-negative breast cancer.

#### 4.7.3 Manufacturing and scalability

All platforms share standard viral transduction for CAR engineering and manufacturing; however, differences lie in adapter production. GCN4-conjugated antibodies can be generated and purified via plasmid vector transfection, enabling high scalability and low cost (89, 134) For DNP, FITC, and biotin labeling of antibodies, non-specific NHS-based chemistry can be performed overnight based on scalable and established workflow (88, 105, 116). However, site-specific small molecule labeling of antibodies requires additional bioengineering of such antibodies to introduce specific amino acids, which will be the site of small molecule conjugation, thus adding complexity to the manufacturing (105). As such, the benefits of site-specific conjugation should be weighed against the cost and manufacturing time. Bioorthogonal click chemistry strategies that metabolically install FITC or DNP-conjugated glycans on tumor cells face short half-life (< 48 hours) requiring frequent dosing (133). Moreover, the seven-step, low-yield synthesis of these conjugates undermines scalability, making the clinical translation of click-chemistry-based adapters very challenging.

#### 4.7.4 Biodistribution and pharmacokinetics

Adapter penetration in the TME is a key factor in the effectiveness of these platforms. Reported antibody-based adapters (trastuzumab, ofatumumab, cetuximab and rituximab) thus far have demonstrated optimal TME infiltration in mouse models (89, 90, 94, 102), yet monoclonal antibodies may have poor penetration in immune privilege sites such as the central nervous system, testis and fibrotic solid tumors with dense TME, constraining biodistribution (135). Pharmacokinetics also vary based on the adapter and its target, further complicating dosing schedule. Camelid or shark-derived nanobodies constitute viable alternatives to antibody-based adapters to enhance TME penetration, due to their small size and capacity to access cryptic epitopes. However, these nanobodies exhibit short tumor retention and rapid clearance (136–138). Hence, both adapter design and administration route will need further optimization to enhance TME infiltration and retention while limiting system clearance.

#### 4.7.5 Head-to-head comparison

Cross-platform comparisons are limited. Nevertheless, one study found that FITC- and GCN4-based adapters yield comparable antitumor activity in TNBC models (99).

## 5 Allogenic adapter-based CAR T cell therapy as a strategy to address autologous adapter-based therapy challenges

Although adapter-based autologous CAR T cells can mitigate antigen escape by targeting multiple TAAs via antibody-labelled adapters, they still face the manufacturing hurdles associated with autologous CAR T cells (18, 20, 21, 139). Allogeneic CAR T cells can overcome these hurdles including the inability to harvest adequate number of T cells to initiate the manufacturing process, and out-of-specification CAR T cells after manufacturing (inadequate CAR expression or viability) especially after cytotoxic chemotherapy where T cell overall fitness can be compromised (140). While, allogeneic adapter-based CAR T cells are not currently being investigated in the clinics, allogeneic monospecific CAR T cells from healthy donors are being extensively tested including in patients who have previously received cytotoxic chemotherapy (7, 9, 22).

As with any allogeneic cell products, allogeneic CAR T cells can induce graft-versus-host disease (GVHD), when αβTCRs on the CAR T cells identify the host tissues as foreign (141, 142). To eliminate the risks of GVHD, multiple strategies including genome editing such as Cytidine Base Editors (CBEs), Clustered Regularly Interspaced Short Palindromic Repeats and CRISPR-associated protein 9 (CRISPR/Cas9), and Transcription activator-like effector nuclease (TALEN) have been utilized to disrupt the T cell receptor alpha constant (TRAC) locus, or Short hairpin RNA (shRNA) to silence TCR mRNA transcripts (23, 143, 144). Alternatively, CAR knock-in at TRAC locus ablates TCR expression and signaling while preserving CAR expression (142, 145). At the protein level, endoplasmic reticulum (ER) retention of the TCR to prevent cell surface expression using an ER retention sequence KDEL-tagged anti-TCR scFv has also been explored (146, 147).

Host immune system can reject allogeneic CAR T cells which may compromise their persistence compared to autologous cell therapies. Therefore, chemotherapy-based (e.g. fludarabine, cyclophosphamide) and anti-CD52 (e.g. alemtuzumab) antibody-based lymphodepletion is usually used to mitigate CAR T rejection, albeit with increased infection risk (146, 148,) (149,) (150,) (151). To reduce the risk of host immune system rejection, β2-microglobulin (B2M) gene knockout, reducing HLA class I expression, is frequently employed in allogeneic CAR T cells (152,) (142,) (22). The resulting HLA-deficient allogeneic CAR T cells can be targeted by host NK cells, leading to CAR T cells rejection, thus HLA-E knock-in can be introduced at the B2M locus to avoid NK cell recognition (153). CD47 overexpression further blunts host innate immune system recognition by NK cells and adaptive T cell responses (7).

Allogeneic CAR T cells with alloreactive gene knockouts can ease manufacturing challenges associated with autologous CAR T cells, while adapter-based autologous CAR T cells can circumvent antigen escape. As such, the combination of these two approaches can be of significant interest and yield a universal “off-the-shelf” CAR T cell therapy. These observations motivate an allogeneic adapter-based CAR T cell platform with alloreactive gene knockout, combining “off-the-shelf” manufacturing with multi-antigen retargeting (**Table 6**), yet to our knowledge, no study has yet reported allogeneic manufacturing of adapter-based CAR T cells from healthy donors.

**Table 6:** Summary of universal CAR T cell strategies.

CAR T cell strategies	Antigen escape	Manufacturing failure
Monospecific Autologous CAR T Cells	Yes	Yes
Allogeneic Monospecific CAR T Cells	Yes	No
Adapter-based Autologous CAR T Cells	No	Yes
Adapter-based allogeneic CAR T Cells	No	No

## 6 Conclusions

Monospecific CAR T cells target a single TAA and are therefore vulnerable to antigen escape through target mutation or downregulation. Developing new monospecific CAR T cells for emerging antigens is slow, risking attrition before efficacy is established. Allogeneic monospecific CAR T cells ease manufacturing challenges but remain susceptible to antigen escape. Adapter-based autologous CAR T cells address antigen escape yet still face manufacturing challenges especially in patients previously treated with cytotoxic chemotherapy. As such, developing a truly “universal” allogeneic adapter-based CAR T cell platform with alloreactive gene knockouts, aiming to pair “off-the-shelf” manufacturing with multi-antigen retargeting can revolutionize CAR T cell therapy against solid tumors. However, further research is needed to reduce immunotoxicity, bolster CAR T cell persistence, trafficking and infiltration into the TME, and optimize the small molecule adapter-based platforms. Integrating allogeneic manufacturing with adapter-based CAR T cell platforms remains untested clinically and demands focused attention to scalability, component optimization, and minimized immunogenicity.
